# Human iPS Cell-Derived Cell Aggregates Exhibited Dermal Papilla Cell Properties in *in vitro* Three-Dimensional Assemblage Mimicking Hair Follicle Structures

**DOI:** 10.3389/fcell.2021.590333

**Published:** 2021-08-02

**Authors:** Masahiro Fukuyama, Aki Tsukashima, Momoko Kimishima, Yoshimi Yamazaki, Hideyuki Okano, Manabu Ohyama

**Affiliations:** ^1^Department of Dermatology, Kyorin University Faculty of Medicine, Tokyo, Japan; ^2^Department of Physiology, Keio University School of Medicine, Tokyo, Japan

**Keywords:** hair follicle, regeneration, epithelial–mesenchymal interactions, dermal papilla, human induced pluripotent stem cells, WNT signaling

## Abstract

Current approaches for human hair follicle (HF) regeneration mostly adopt cell-autonomous tissue reassembly in a permissive murine intracorporeal environment. This, together with the limitation in human-derived trichogenic starting materials, potentially hinders the bioengineering of human HF structures, especially for the drug discovery and treatment of hair loss disorders. In this study, we attempted to reproduce the anatomical relationship between an epithelial main body and the dermal papilla (DP) within HF *in vitro* by three-dimensionally assembling columnarly molded human keratinocytes (KCs) and the aggregates of DP cells and evaluated how HF characteristics were reproduced in the constructs. The replaceability of human-induced pluripotent stem cell (hiPSC)-derived DP substitutes was assessed using the aforementioned reconstruction assay. Human DP cell aggregates were embedded into Matrigel as a cluster. Subsequently, highly condensed human KCs were cylindrically injected onto DP spheroids. After 2-week culture, the structures visually mimicking HFs were obtained. KC-DP constructs partially reproduced HF microanatomy and demonstrated differential keratin (KRT) expression pattern in HFs: KRT14 in the outermost part and KRT13, KRT17, and KRT40, respectively, in the inner portion of the main body. KC-DP constructs tended to upregulate HF-related genes, *KRT25*, *KRT33A*, *KRT82*, *WNT5A*, and *LEF1*. Next, DP substitutes were prepared by exposing hiPSC-derived mesenchymal cells to retinoic acid and subsequently to WNT, BMP, and FGF signal activators, followed by cell aggregation. The resultant hiPSC-derived DP substitutes (iDPs) were combined with KCs in the invented assay. KC-iDP constructs morphologically resemble KC-DP constructs and analogously mimicked KRT expression pattern in HF. iDP in the constructs expressed DP-related markers, such as vimentin and versican. Intriguingly, KC-iDP constructs more intensely expressed *KRT33A*, *KRT82*, and *LEF1*, which were stepwisely upregulated by the addition of WNT ligand and the mixture of WNT, SHH, and EDA signaling activators, supporting the idea that iDP exhibited biological properties analogous to DP cell aggregates in the constructs *in vitro*. These preliminary findings suggested the possibility of regenerating DP equivalents with *in vitro* hair-inductive capacity using hiPSC-derived cell composites, which potentially reduce the necessity of human tissue-derived trichogenic cell subset and eventually allow xeno-free bioengineering of human HFs.

## Introduction

The hair follicle (HF) is a multifunctional mammalian skin appendage, providing a physical barrier against external insults, facilitating thermoregulation, and transmitting tactile sense (Stenn and Paus, 2001; Schneider et al., 2009; Nagao et al., 2012; Zimmerman et al., 2014). In the case of humans, hairstyles greatly influence one’s appearance and therefore vast demand exists for the treatment of hair loss disorders (Saed et al., 2017; Ohyama, 2019). Establishment of a methodology to experimentally regenerate human HFs is of major significance in the management of hair loss conditions as a way of preparing therapeutic materials for hair transplantation and supplying an experimental platform for drug discovery (Ohyama, 2019). Considering the structural and functional complexity of HF for a small dimension miniorgan, HF bioengineering, especially that achieved *in vitro*, may provide a platform for the regeneration of other large-sized tissue or organs.

The HF is a cylindric structure composed of the epithelial main body consisting of keratinocytes (KCs) including stem cells and the mesenchymal structures; the dermal papilla (DP), a specialized hair-inductive dermal cell aggregate located at the bottom (bulb) of the HF, and the dermal sheath (DS) surrounding the main body (Yang and Cotsarelis, 2010; Ohyama, 2019). The HF continuously self-renews throughout life *via* the hair cycle (Stenn and Paus, 2001). Both HF morphogenesis and regeneration are enabled by well-orchestrated epithelial–mesenchymal interactions (EMIs) *via* biological signaling pathways, such as WNT, BMP, Sonic hedgehog (SHH), and Ectodysplasin A (EDA) pathways (St-Jacques et al., 1998; Millar, 2002; Zhang et al., 2009; Mikkola, 2011; Sennett and Rendl, 2012; Ohyama, 2019). To achieve successful HF regeneration, sufficient folliculogenic EMIs need to be elicited and maintained between receptive KCs and inductive DP (Millar, 2002; Sennett and Rendl, 2012; Ohyama, 2019).

To date, experimental HF regeneration has been attempted mainly by co-transplantation of KCs and trichogenic DP cells into an *in vivo* environment represented by the intracorporeal spaces of immunodeficient mice (Weinberg et al., 1993; Zheng et al., 2005; Ehama et al., 2007; Kobayashi et al., 2010). The transplanted cells evoke EMIs and cell-autonomously reassemble HF structures, which is constantly successful for murine cells and has recently been stabilized for human cells (Thangapazham et al., 2014a, b; Yoshida et al., 2019). Obviously, use of xeno-free environment is far more preferable considering the direct application of regenerated structures for regenerative medicine purposes (e.g., transplantation to hair loss areas). Adopting 3D-printing technology to precast HF-shape cavities in vascularized collagen gel that were filled with DP cell (DPC) aggregates and KCs, Abaci et al. recently bioengineered HF structures from human cells with the hair shaft diameter fairly comparable to that of human intermediate-vellus hairs, opening the door for mass production of bioengineered human HFs for regenerative medicine and pharmaceutical investigations (Abaci et al., 2018). At the same time, the approach requires advanced technologies and still not readily available for most laboratories. For drug discovery, a simpler platform allowing the regeneration of HF-like structures partially recapitulating pivotal HF characteristics would be beneficial.

Efficiency of experimental human HF regeneration in the abovementioned approaches can also be influenced by the biological properties of starting materials; for KCs, those derived from neonatal or juvenile individuals (Ehama et al., 2007; Thangapazham et al., 2014a), and for dermal cells, DP or DS cells expressing high levels of hair inductive markers (McElwee et al., 2003; Driskell et al., 2009; Ohyama et al., 2012; Higgins et al., 2013) have been considered to be preferable. Of note, preparation of DP cells with potent trichogenicity has been recognized to be a gold standard for such application (Yang and Cotsarelis, 2010; Ohyama, 2019). However, HF-derived DP cells collectable from hair loss patients are limited. In addition, DP lose their intrinsic properties after *in vitro* expansion, representing another major hurdle in human HF regeneration (Weinberg et al., 1993; Rendl et al., 2008; Ohyama et al., 2012; Higgins et al., 2013; Ohyama, 2019). Establishment of alternative material sources of DP cells should resolve such difficulties.

Human-induced pluripotent stem cells (hiPSCs) hold promise as a substituting cell source (Ohyama and Okano, 2014). Past studies demonstrated that hiPSCs could be differentiated into KC precursors or DP substituting cells and contribute to regeneration of HF-like structures in *in vivo* HF reconstitution assays (Veraitch et al., 2013, 2017). Imperfectness of regenerated structures and low efficiency of reproduction highlight the necessity of further improvements. In addition, whether hiPSC-derived cells exhibit analogous trichogenicity *in vitro* remains unknown.

In this study, we attempted to establish a simple assay in which highly condensed human KCs and the aggregates of DP cells were three-dimensionally assembled *in vitro* mimicking their anatomical association in HFs and cultured for 2 weeks. Histological and molecular biological analyses of regenerated structures were conducted to assess the resemblance of regenerated structures to HFs. hiPSC-derived mesenchymal cells were exposed to retinoic acid and subsequently to WNT, BMP, and FGF signal activators, followed by cell aggregation, expecting to elicit DP properties. The replaceability of hiPSC-derived DP substituting cell aggregates with DPs was assessed using the assay developed. Effects of folliculogenic signaling pathway activation on the regenerated structures by WNT, SHH, and EDA agonists were also evaluated.

## Materials and Methods

### Preparation of KCs, DP Cells, and Fibroblasts

Human KCs and DP cells were purchased from CELLnTECH Advanced Cell systems AG (Bern, Switzerland) and PromoCELL (Heidelberg, Germany), respectively, or isolated from adult human scalp samples obtained from surgery of benign scalp tumors as previously described. Human fibroblasts were purchased from PromoCELL (Heidelberg, Germany). All donors provided written informed consent in accordance with the Declaration of Helsinki. KCs at passages 3–6, fibroblasts at passage 5, and DP cells at passages 2–4 were used. KCs, fibroblasts, and DP cells were, respectively, cultured in CnT-PR (CELLnTECH Advanced Cell systems AG, Bern, Switzerland), Dulbecco’s Modified Eagle’s Medium (DMEM; Sigma-Aldrich, St. Louis, MO, United States) containing 10% fetal bovine serum (FBS; Biowest, Nuaillé, France), and follicle dermal papilla cell medium (DP medium; PromoCELL, Heidelberg, Germany) at 37°C in a 5% CO_2_ prior to HF reconstruction assay. Culture medium was changed every 2 days.

### Generation of Cell Aggregates From hiPSC-Derived DP Substituting Cells

hiPSC-derived DP substituting cells (iDPSCs) were generated following the previously published protocol with minor modifications (Veraitch et al., 2017). Three lines of hiPSCs generated by retroviral introduction of four Yamanaka factors into dermal fibroblasts [201B7 (Takahashi et al., 2007) and WD39 (Imaizumi et al., 2012)] or by gene delivery using a single, synthetic self-replicating VEE-RF RNA replicon into endothelial progenitor cells [RPChiPS771-2 (ReproCELL Inc., Yokohama, Japan)] were maintained on feeder-free culture dish filled with StemFit^TM^ (AJINOMOTO, Tokyo, Japan) following the manufacturer’s protocol. For hiPSC-derived mesenchymal stem cell-like cell (iMC) induction, 6.5 × 10^4^ hiPSCs/well in a six-well plate were placed onto a humanized substrate (CELLstart CTS; Life Technologies, Carlsbad, CA, United States). When a well was approximately 80% confluent, mesenchymal cell induction was initiated using Stempro MSC-SFM CTS (Life Technologies, Carlsbad, CA, United States) for 9–11 days. iMCs were then incubated in DMEM (Sigma-Aldrich, St. Louis, MO, United States) containing 10% FBS (Biowest, Nuaillé, France) with 10 μM all-trans retinoic acid (Sigma-Aldrich, St. Louis, MO, United States) for 4 days, and subsequently in DP cell-activating culture (DPAC) medium (Ohyama et al., 2012); 10% FBS-DMEM supplemented with 1 μM 6-bromoindirubin-3’-oxime (Sigma-Aldrich, St. Louis, MO, United States), 20 ng/ml bFGF (Peprotech, Rocky Hill, NJ, United States), and 200 ng/ml human recombinant BMP2 (R&D Systems, Minneapolis, MN, United States) for 7 days to obtain iDPSCs.

### Preparation of DP Cell or iDPSC Aggregates

A total of 1 × 10^6^ DP cells or iDPSCs were suspended in DP medium and stained with CellBrite^TM^ Orange Cytoplasmic Membrane Dye (Biotium, Hayward, CA, United States) following the manufacturer’s protocol (Veraitch et al., 2017). After washing three times with DP medium, 5 × 10^3^ DP cells or iDPSCs were seeded into each well of non-cell binding 96-well plate (Thermo Fisher Scientific, Rockford, IL, United States) containing DP medium. The plates were centrifuged at 500 rpm for 2 min and incubated at 37°C, 5% CO_2_ with medium changes every 5 days to form DP-like cell aggregates.

### Reconstitution of HF-Like Structure *in vitro*

**FIGURE 1:**
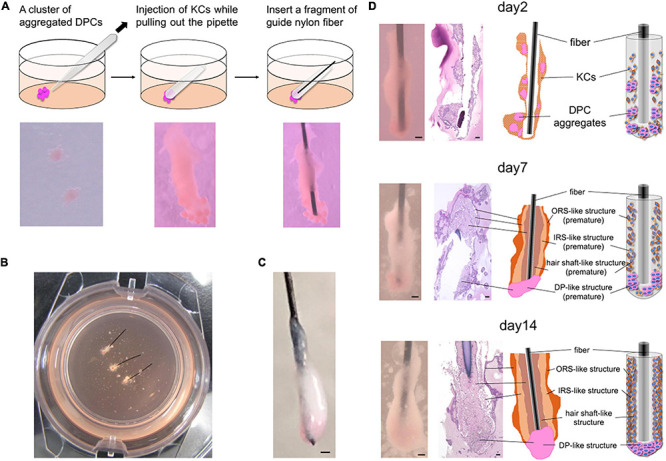
*In vitro* 3D regeneration of KC-DP constructs and time course changes in their intratissue architecture to mimic representative HF structural features during 2-week culture. **(A)** The schema of the reconstruction steps. DP (or iDPSC) aggregates were placed in Matrigel to form clusters. Condensed cylindrical KC structure was placed in close contact with DP aggregates. A nylon fiber was inserted as a structural supporter and guide for histological sectioning. **(B)** KC-DP constructs in Matrigel after 1-week culture. **(C)** KC-DP constructs harvested after 2-week culture. The approximated size was analogous to that of human HF. **(D)** Time course change in the intratissue architecture of KC-DP constructs at 2 days, 1 week, and 2 weeks. The constructs gradually reorganize their structure and formed densely gathered DP aggregates and multiple KC layers (ORS-, IRS-, and hair shaft-like) sketchily mimicking human HFs, especially the bulb and suprabulbar portion. DP, dermal papilla; DPC, dermal papilla cells; KC, keratinocyte; IRS, the inner root sheath; ORS, the outer root sheath. Scale bar: 200 μm.

Using dissection microscopes and a micropipette, 12 DP cell or iDPSC aggregates were placed in a group within 3 ml of Matrigel matrix (CORNING, Corning, NY, United States) in a cell culture insert for a six-well cell culture plate (Gibco, Rockford, IL, United States). Subsequently, 5 μl of highly condensed human KCs (23.4 × 10^6^ on average per experiment) suspended in 500 μl of CnT-PR was cylindrically injected onto aggregated DP cell or iDPSC spheres embedded in Matrigel matrix, while pulling a micropipette outward, to form a structure mimicking HF architecture (Figure 1A). A fragment of guide nylon wire (BEAR Medical, Ibaraki, Japan) was inserted within KC composites as a scaffold (Figure 1A). The Matrigel matrix containing HF-like structures was submerged in the 1:1 mixture of CnT-PR and Amnio MAX-C100 (Gibco, Rockford, IL, United States) and incubated at 37°C, 5% CO_2_ for 2 weeks. The medium was changed on alternate days.

### Stimulation of Folliculogenic Signaling by Agonists

To activate HF morphogenesis associated WNT, SHH, and EDA signaling pathways in HF-like constructs, 2 μM CHIR99021 [the glycogen synthase kinase (GSK) 3β inhibitor (a WNT signal agonist) (Ring et al., 2003); Cayman Chemical, Ann Arbor, MI, United States], 2 μM purmorphamine [the Smo receptor agonist (a SHH signal agonist) (Sinha and Chen, 2006); FUJIFILM Wako Pure Chemical Co., Osaka, Japan], and 10 ng/ml EDA (recombinant ectodysplasin A1; R&D Systems, Minneapolis, MN, United States) were singly or jointly added to culture medium at the generation of HF-like structure and continued during 2-week culture.

### Quantitative Reverse Transcription-Polymerase Chain Reaction

Total RNA was extracted using the RNeasy Mini Kit (Qiagen, Hilden, Germany) and cDNA was synthesized using the Superscript III First Strand Synthesis SuperMix (Invitrogen, Carlsbad, CA, United States) according to the manufacturers’ instructions. All the used primer sequences are shown in Supplementary Table 1. Real-time reverse transcription-polymerase chain reaction (RT-PCR) analyses were conducted using the PowerUp^TM^ SYBR^TM^ Green PCR Master Mix and the StepOne Real-Time PCR system (Applied Biosystems, Foster City, CA, United States). Cycling conditions consisted of an initial activation of 2 min at 95°C, then 40 cycles of denaturing at 95°C for 15 s, and annealing and extension at 60°C for 1 min. Messenger RNA expression levels were normalized to β-actin expression level adopting the ΔCT method and calculated based on 2^–ΔCT^.

### Histological and Immunohistochemical Analysis

Harvested HF-like constructs were fixed by 10% formalin and embedded in paraffin. For histological staining, paraffin-embedded tissue sections were deparaffinized and stained with Carrazzi’s hematoxylin and counterstaining with eosin before mounted in malinol. For immunohistochemical analyses, the deparaffinized sections were pretreated in an autoclave (121°C, 5–10 min, pH 6), blocked endogenous peroxidase by 3% hydrogen peroxide at RT for 10 min, and incubated overnight at 4°C with the following primary antibodies: mouse anti-human cytokeratin 13 (KRT13) monoclonal antibody (Ks13.1; 1:100; PROGEN, Heidelberg, Germany), mouse anti-human cytokeratin 14 (KRT14) monoclonal antibody (LL002; 1:500; Abcam, Cambridge, MA, United States), rabbit anti-human cytokeratin 17 (KRT17) polyclonal antibody (ab53707; 1:200; Abcam, Cambridge, MA, United States), mouse anti-human cytokeratin 19 (KRT19) monoclonal antibody (Ks19.1; 1:200; Biocare Medical, Pacheco, CA, United States), mouse anti-human hair cortex cytokeratin/K40 (KRT40) monoclonal antibody (AE13; 1:50; Abcam, Cambridge, MA, United States), rabbit anti-human cytokeratin 75 (KRT75) polyclonal antibody (ab254740; 1:200; Abcam, Cambridge, MA, United States), rabbit anti-human GATA binding protein 3 (GATA3) polyclonal antibody (GTX109654; Gene Tex, Irvine, CA, United States), rabbit anti-human dickkopf 4 polyclonal antibody (DKK4; 1:100; Abcam, Cambridge, MA, United States), rabbit anti-human sex determining region Y-box 2 (SOX2) monoclonal antibody (ab92494; 1:100; Abcam, Cambridge, MA, United States), rabbit anti-human lymphoid enhancer binding factor 1 (LEF1) monoclonal antibody (ab137872; 1:100; Abcam, Cambridge, MA, United States), mouse anti-human vimentin (VIM) monoclonal antibody (V9; 1:1; DAKO, Glostrup, Denmark), and mouse anti-human smooth muscle actin (SMA) monoclonal antibody (1A4; 1:1; DAKO, Glostrup, Denmark). For versican (VCAN) staining, the sections were incubated 2 h at RT with rabbit anti-human VCAN polyclonal antibody (versican V0, V1 neo; 1:100; Thermo Fisher Scientific, Rockford, IL, United States). After washing by tris-buffered saline, samples were incubated with secondary anti-mouse or rabbit antibodies (ENVISION Dual link system; DAKO, Glostrup, Denmark) for 30 min at RT. Subsequently, the sections were visualized by incubation with 3-amino-9-ethylcarbazole (AEC; DAKO, Glostrup, Denmark) and nuclei were counterstained by Mayer’s hematoxylin.

### Alkaline Phosphatase Staining

Frozen tissue sections were fixed with acetone for 10 min at 4°C. After washing with tris-buffered saline for 5 min, sections were exposed to 1 mg/ml alkaline phosphatase (ALP) Fast Blue RR salt (F0500; Sigma-Aldrich, St. Louis, MO, United States) in 0.2 mol/L 2-amino-2-methyl-1,3-propanediol-HCl buffer solution (pH 8.6) (A9754; Sigma-Aldrich, St. Louis, MO, United States) containing 0.05 mg/ml naphthol AS-MX phosphate disodium salt phosphatase substrate (N5000; Sigma-Aldrich, St. Louis, MO, United States) and 2% N,N-dimethylformamide (D4254; Sigma-Aldrich, St. Louis, MO, United States) for 45 min at 37°C. Subsequently, sections were counterstained by nuclear fast red (Muto Chemical KK, Tokyo, Japan) for 10 min.

### Immunofluorescent Analysis

The samples were embedded in OCT and kept at -80°C before sectioning. The cryopreserved sections were defrosted, fixed with acetone at 4°C for 10 min, washed twice with PBS, and incubated with mouse anti-human vimentin (VIM) monoclonal antibody (V9; 1:1; DAKO, Glostrup, Denmark) for 30 min at RT, washed, and then incubated with Alexa Flour 488 goat anti-mouse IgG (H + L) polyclonal secondary antibody (A11029; 1:100; Invitrogen, Carlsbad, CA, United States) for 40 min at RT. The nuclei were stained by 4’,6-diamidino-2-phenylindole (1:1,500; Dojindo Molecular Technologies, Inc., Kumamoto, Japan). After washing, specimens were mounted with PermaFluor Aqueous Mounting Medium (Thermo Fisher Scientific, Rockford, IL, United States). The images were obtained using BZ-X710 (KEYENCE, Osaka, Japan).

### Statistical Analysis

Statistical analysis in this study was performed by Wilcoxon signed-rank test using SPSS version 25 (IBM, Armonk, NY, United States). A *p*-value of less than 0.05 was considered as statistically significant.

## Results

### Normal KC and DP-Derived Constructs Reorganized Intratissue Architecture to Mimic Representative HF Structural Features During 2-Week Culture

Immediately after DP aggregates and condensed KCs were placed in Matrigel to reproduce their anatomical locations in HFs, individual cell components were in close contact with each other but clearly distinguishable (Figure 1A). In our pilot study, the constructs were generated and cultured without any auxiliary structural material, leading to the loss of distinct morphological characteristics and difficulty in preparation of histological sections under the absence of a direction indicator (Supplementary Figure 1). Being inspired by the work of Toyoshima et al. (2012), a nylon fiber was inserted into the main body of the KC-DP construct, which sustained the structural integrity at the time of reconstitution (Figures 1A–C).

During 2-week culture, the constructs grew and reformed gross morphology to a cudgel-like appearance with smoother surface visually mimicking human HFs, especially the suprabulbar (stem) and bulb portion (Figures 1B–D). Histological analysis of KC-DP constructs is technically challenging because of their smallness and fragility and illustrations were provided for each panel (Figure 1D). In 2-day cultured constructs, DP aggregates remained unfused, distributing around the end of the structures. A week later, the fundamental morphology was unchanged; however, the diameter of cylindrical body increased. Interestingly, DP aggregates seemed to relocate themselves to more densely distribute at the end portion. After 2 weeks, the end portion of the reconstructed structure usually enlarged resembling the bulb portion of the HF (Figures 1C,D). The orange-tinted end reflects dense accumulation of dye-stained DP aggregates, suggesting cell-autonomous self-reorganization (Figure 1D). The main body of KC-DP constructs consisted of major components subdivided by nylon fibers, individually consisting of three histologically distinct KC layers with differential eosin staining intensities (Figure 1D). This architecture resembled the arrangement of the outer and inner root sheath (ORS and IRS), and the hair shaft within HF (Figure 1D and Supplementary Figure 2). Human fibroblasts could be aggregated to form spheroid structure resembling DP aggregates (Supplementary Figure 3A); however, constructs generated with KC and fibroblast aggregates failed to form KC layers analogous to those observed in KC-DP constructs (Supplementary Figure 3B).

When KC-DP constructs cultured for 4 weeks were compared to those cultured for 2 weeks, morphologically analogous structures were formed; however, dyskeratotic cell-like changes with homogenous eosinophilic cytoplasm were observed in the constructs cultured for 4 weeks (Supplementary Figure 4).

These findings suggested that KC-DP constructs reorganized intratissue architecture to mimic representative HF structural features, especially those of the suprabulbar to bulb portion, during 2-week culture, possibly *via* trichogenic EMI between KC and DP components.

### KC-DP Constructs Sketchily Reproduced Compartmental HF Characteristics in Immunohistochemistry

The HF is biochemically characterized by its distinct keratin and mesenchymal marker expression profiles. In normal human HFs, keratin (KRT) 13 is expressed in IRS, while KRT14 is detected in ORS. KRT17 is expressed in the inner aspect of ORS and KRT19 is diffusely detectable in ORS from the bulge to bulb. Immunoreactivity of KRT40, a representative hair hard keratin, is specifically observed in the hair shaft. VIM is a global mesenchymal marker expressed in the dermal portion of the HF. Expression levels of VCAN and SMA have been reported to correlate with DP and DS cell properties, including hair inductive property (Jahoda et al., 1991; Kishimoto et al., 1999; Kishimoto et al., 2000). These markers are preferentially expressed in the proximal portion of the dermal sheath and partially in the DP (Ohyama et al., 2010) (Supplementary Figure 5).

**FIGURE 2:**
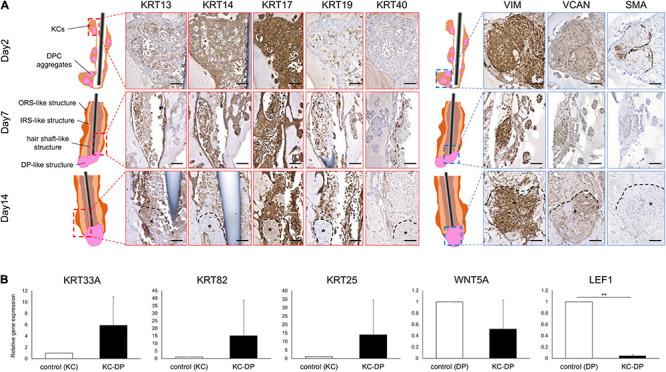
Immunohistochemical and gene expression analysis of HF marker expression in KC-DP construct. **(A)** Time course immunohistochemical analyses revealed that the constructs regenerated with keratinocytes and dermal papilla cells (KC-DP) gradually recapitulated hair follicle (HF) marker expression patterns resembling those in human HF (see Supplementary Figure S6) during 2-week culture. KRT13 is expressed in the inner portion, while KRT14 is detected in the outer portion. KRT17 is diffusedly expressed in the main body, while KRT19 gradually became detectable in the inner and outermost portion. Linear immunoreactivity of KRT40 extended from DP. VIM, VCAN, and SMA were detected in DP. *: DP aggregates. **(B)** The expression levels of HF-related genes in KC-DP constructs (Mean ± SEM; *n* = 5, triplicate for each experimentation; **p < 0.01, Wilcoxon signed-rank test). DP, dermal papilla; DPC, dermal papilla cells; KC, keratinocyte; KRT, keratin; VIM, vimentin; VCAN, versican; SMA, smooth muscle actin. Scale bar: 200 μm.

Time course immunohistochemical analyses suggested that KC-DP constructs gradually recapitulated microanatomical keratin and mesenchymal marker expression patterns of normal human HFs during 2-week culture (Figure 2A). In 2-day constructs, KRT13 demonstrated a weak and diffuse expression pattern in the KC compartment. However, KRT13 expression was confined to the inner layer of 1-week constructs, which became more intense and broader in 2-week constructs. At the beginning of the culture, KRT14 diffusely expressed in KC compartment in 2-day constructs, which localized to the layer next to that expressing KRT13, analogous to the HF. KRT17 was rather non-specifically expressed from the beginning of the assay, while KRT19 expression increased in the KRT14 expressed area similarly to normal HFs (Fukuyama et al., 2017). Interestingly, KRT40 expression, which is specific to the hair shaft and highlighted by AE13-positive immunoreactivity (Supplementary Figure 5), was initially negative but started to become detectable in KC strands located adjacent to the KRT13 expression area extending from the interface between DP aggregates after 1-week culture, which was maintained at 2 weeks.

VIM and VCAN were consistently detected in DP aggregates, providing a useful marker to distinguish dermal cells from KCs and implying that DP properties were maintained to some extent in the constructs (Figure 2A). The expression level of SMA, which rather represents a dermal sheath marker than that of DP (Supplementary Figure 5), gradually decreased in DP aggregates during the assay (Figure 2A).

These findings suggested that KC-DP constructs sketchily reproduced biochemical characteristics of individual HF compartments in a time-dependent manner during the 2-week assay.

### Reconstituted HF-Like Structures Upregulated HF-Related Epithelial Genes While Downregulating WNT Signaling Genes

When compared to cultured human KCs, KC-DP constructs tendentiously upregulated *KRT33A* (hair shaft cortex keratin), *KRT82* (hair shaft cuticle keratin), and *KRT25* (IRS keratin) (Langbein et al., 2006; Schweizer et al., 2007), respectively, by 5.9-fold (*p* = 0.095), 15.1-fold (*p* = 0.095), and 14-fold (*p* = 0.095) on average. In contrast, representative HF-related WNT signaling genes *WNT5A* (DP marker) and *LEF1* (DP > HF-epithelial marker) were downregulated in KC-DP constructs compared to DPCs by 1.9-fold (*p* = 0.095) and 25-fold (*p* < 0.01), respectively (Figure 2B).

These observations suggested that folliculogenic EMIs between KC and DP components were elicited in the constructs and that the magnitude of EMI was insufficient because of the loss of DP properties *in vitro*.

### Activation of WNT, SHH, and EDA Signaling Improved HF-Related Marker Expression in KC-DP Constructs

**FIGURE 3:**
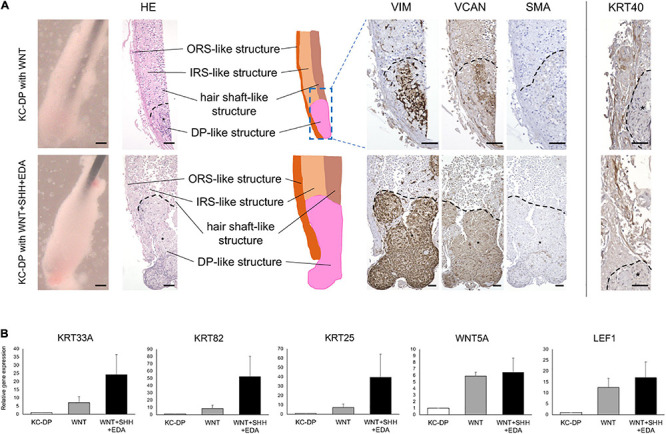
Activation of WNT, SHH, and EDA signaling improved HF-related marker expression in KC-DP constructs. **(A)** Addition of the activators of signaling pathways involved in HF morphogenesis did not evidently ameliorate immunohistochemically detected HF marker expression in KC-DP constructs. *DP aggregates. **(B)** HF-related gene expression levels analyzed by qRT-PCR in KC-DP constructs tended to be increased by a WNT agonist (WNT) treatment, which tended to be further enhanced by the treatment by the mixture of agonists (WNT + SHH + EDA) (Mean ± SEM; *n* = 3, triplicate for each experimentation; Wilcoxon signed-rank test). KC, keratinocyte; DP, dermal papilla, Scale bar: 200 μm.

WNT signaling pathway has been reported to play key roles in HF morphogenesis and regeneration (Millar, 2002; Sennett and Rendl, 2012). Addition of WNT signaling agonists has been shown to sustain or ameliorate impaired intrinsic properties of human cultured DP cells (Ohyama et al., 2012; Soma et al., 2012). When exposed to a WNT agonist, CHIR99021 (Ring et al., 2003), KC-DP constructs did not markedly change their morphology. VIM, VCAN, and SMA immunoreactivity were maintained; however, gene expression analysis detected that HF-related genes, *KRT33A*, *KRT82*, *KRT25, WNT5A*, and *LEF1*, tended to be upregulated by the WNT agonist (Figures 3A,B).

Sonic hedgehog (SHH) and EDA signaling pathways are also crucial in folliculogenic EMIs (St-Jacques et al., 1998; Zhang et al., 2009; Mikkola, 2011). A recent study suggested that WNT, SHH, and EDA signaling agonists synergistically enhanced HF-related gene expression in cultured three-dimensional skin equivalent (Fukuyama et al., 2020). Based on this observation, the mixture of WNT, SHH, and EDA agonists was supplemented to the culture medium during the assay. Despite the fact that KRT40 immunoreactivity seemed to be mildly intensified, an increase in immunoreactivity of VIM, VCAM, and SMA was not evident in the mixture-treated KC-DP constructs. In contrast, further enhancement of HF-related keratin and WNT gene expression levels was noted when compared to those in untreated and WNT agonist-treated KC-DP constructs (Figures 3A,B).

Despite the fact that the extent of amelioration was moderate by the aforementioned combination, these findings favored the combined use of folliculogenic signaling activators for the quality improvement of KC-DP constructs.

### iDPSC Aggregates Partially Recapitulated DP Properties*in vitro*

**FIGURE 4:**
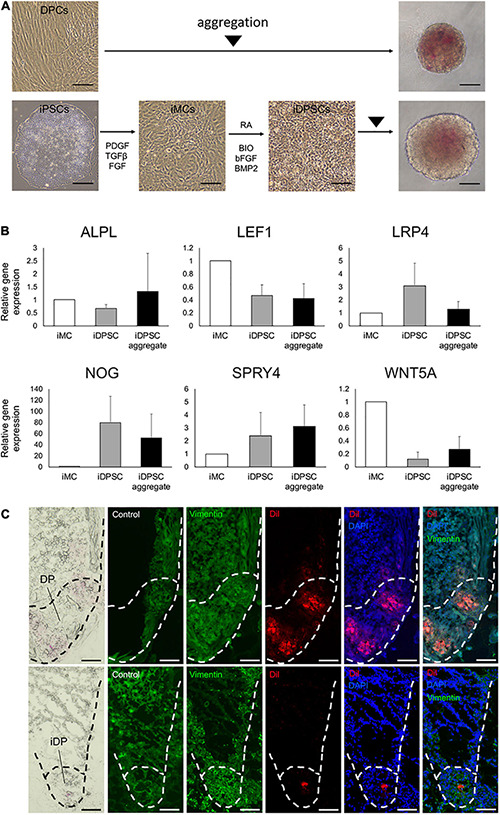
Generation of hiPSC-derived dermal papilla substituting cell aggregates. **(A)** Aggregation of dermal papilla cells (DPCs) and the stepwise induction of human-induced pluripotent stem cell (hiPSC)-derived DP substituting cells (iDPSCs) from hiPSC-derived mesenchymal cells (iMCs) and the generation of iDPSC aggregates (iDPs). Scale bar: 100 μm. **(B)** Change in DP marker gene expression analyzed by qRT-PCR during iDP aggregate preparation from iMCs. (Mean ± SEM; *n* = 3, triplicate for each experimentation; Wilcoxon signed-rank test). **(C)** Immunofluorescent images of KC-DP (upper panels) and KC-iDP constructs (lower panels). Low dyeability to cytoplasmic membrane dyes distinguished iDPs from DPs. Scale bar: 200 μm.

A past study reported that hiPSC-derived DP substituting cells (iDPSCs) share some biological characteristics of human intact DP cells, including hair inductive capacity, as demonstrated by *in vivo* HF reconstruction assay (Veraitch et al., 2017). Use of iDPSC aggregates for the replacement of DP aggregates in KC-DP constructs would be a reasonable approach to assess iDPSC properties *ex vivo*. Accordingly, three lines of hiPSCs were, respectively, induced into mesenchymal cells (iMCs) and further differentiated into iDPSCs and subsequently aggregated based on the previously established protocol (Ohyama et al., 2012) (Figure 4A).

Spheroid formation adopting low cell-binding plate yielded DP and iDPSC aggregates with respective efficiency. Non-induced hiPSCs failed to form spheroids, suggesting that deviation into mesenchymal lineage is required in this methodology (Supplementary Figure 6). DP and WD39-hiPSC-derived iDPSCs successfully formed spheroid structures with a smooth surface (Figure 4A). Unexpectedly, 201B7-hiPSC- or RPC-hiPS771-2-derived iDPSC gave rise to cells that formed less compactly or irregularly aggregated structures (Supplementary Figure 7). Thus, WD39-hiPSC-derived iDPSC aggregates, hereafter termed as iDPs, were predominantly used for downstream experimentations.

A previous study reported the upregulation of selected DP markers during iDPSC induction from iMCs using the same protocol, but hiPSCs were maintained on feeders (Veraitch et al., 2017). In the present study adopting feeder-free hiPSCs, DP biomarkers, *NOG*, *SPRY4*, and *LRP4*, tended to be upregulated in iDPSCs compared to iMCs. Other DP or WNT signaling markers, *ALPL*, *WNT5A*, and *LEF1* (Ohyama et al., 2012), were downregulated in iDPSCs when compared to iMCs (Figure 4B). The expression levels of *ALPL*, *SPRY4*, and *WNT5A* were moderately restored by aggregating iDPSCs.

iDPs aggregates were able to locate at the bottom of HF-like structures during 2-week culture similarly to DP aggregates in KC-DP constructs. iDPs were less intensely stained with CellBrite^TM^ dye than DP aggregates and distinguishable (Figure 4C).

### KC-iDP Constructs Reproduced Architectural/Biochemical Characteristics and Gene Expression Profile of KC-DP Constructs

**FIGURE 5:**
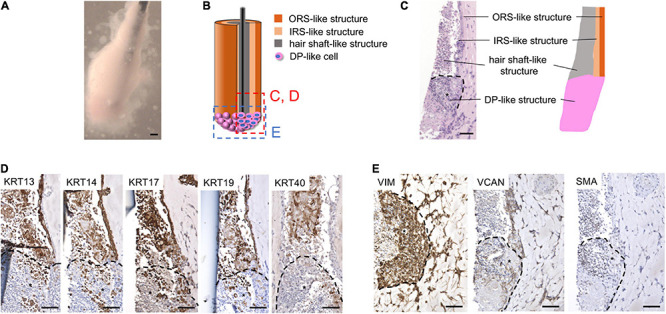
KC-iDP constructs reproduced architectural/biochemical characteristics and gene expression profile of KC-DP constructs. **(A)** Morphology of KC-iDP constructs. Scale bar: 200 μm. **(B)** The schema illustrating the intratissue structure of KC-iDP constructs. **(C)** Histological characteristics of KC-iDP constructs resembling those of KC-DP constructs (see Figure 2). *: iDP aggregates. Scale bar: 200 μm. **(D)** Immunohistochemical analysis revealed that HF keratin distribution was almost indistinguishable between KC-DP and KC-iDP constructs (see Figure 2). KRT40-positive cells rearranged in the center of the main body mimicking the hair shaft without being splitted by a nylon fiber (the rightmost panel). *: iDP aggregates. Scale bar: 200 μm. **(E)** Immunoreactivities for mesenchymal markers in KC-iDP constructs. *: iDP aggregates. Scale bar: 200 μm; DP, dermal papilla; KC, keratinocyte; KRT, keratin; HF, hair follicle; iDP, human induced pluripotent cell-derived dermal papilla substituting cell aggregate; IRS, inner root sheath; ORS, outer root sheath; SMA, smooth muscle actin; VCAN, versican; VIM, vimentin.

When combined with cylindrically condensed KCs, iDPs formed structures morphologically analogous to KC-DP constructs (Figure 5A). Histologically, KC-iDP constructs reproduced architectural characteristics of KC-DP constructs, partially mimicking HF structures (Figures 5B,C).

Immunohistochemical examination demonstrated that KC-iDP constructs expressed HF-related markers in the same manner as KC-DP constructs (Figure 5D). Of note, intense immunoreactivity of hair shaft keratin, KRT40, as detected by AE13 monoclonal antibody, was observed in some KC-iDP constructs (Figure 5D). Expression levels of DP markers, VIM, VCAN, and SMA, in iDP aggregates were also comparable to those in KC-DP constructs (Figures 2C, 5E).

**FIGURE 6:**
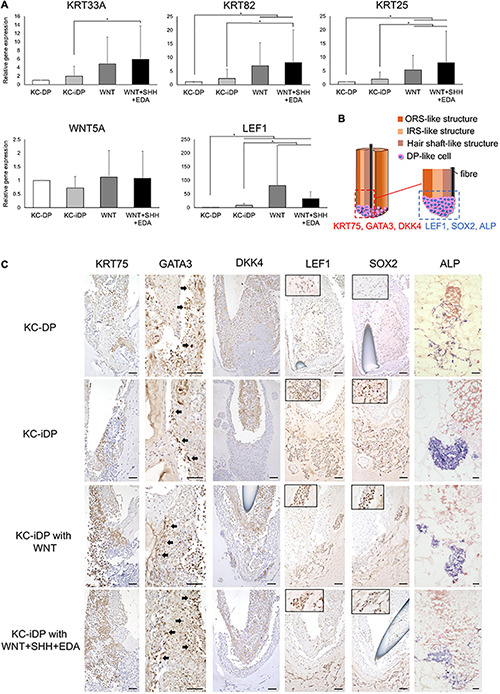
KC-iDP constructs responded to WNT, SHH, and EDA agonists to upregulate HF-related markers. **(A)** A WNT agonist increased HF keratin gene and *LEF1* expression in KC-iDP constructs, which was further enhanced by the simultaneous activation of WNT, SHH, and EDA signaling pathways (Mean ± SEM; *n* = 5, triplicate for each experimentation; *p < 0.05, Wilcoxon signed-rank test). **(B)** The schema illustrating the intratissue distribution of examined hair follicle markers within KC-iDP constructs. **(C)** Immunoreactivities for hair follicle epithelial (KRT75, GATA3, and DKK4) and mesenchymal (LEF1, SOX2, and ALP) markers in non-treated, WNT agonist-treated, and triple agonist-treated KC-iDP constructs in comparison to those in KC-DP constructs. KRT75, SOX2, and LEF1 immunoreactivities were more intense in KC-iDP constructs, when compared to those in KC-DP constructs. Scale bar: 200 μm. ALP, alkaline phosphatase; DKK4, dickkopf 4; DP, dermal papilla; GATA3, GATA binding protein 3; iDP, keratinocyte-human induced pluripotent cell-derived dermal papilla substituting cell aggregate; KC, keratinocyte; KRT, keratin; LEF1, lymphoid enhancer binding factor 1; SOX2, sex determining region Y-box 2.

Intriguingly, hair shaft and IRS keratin genes, *KRT25*, *KRT33A*, and *KRT82*, were more intensely expressed in KC-iDP constructs than in KC-DP constructs (Figure 6A). *WNT5A* was almost equally expressed between KC-iDP and KC-DP constructs, while *LEF1* was upregulated by 8.7-fold in KC-iDP constructs (Figure 6A).

These findings suggested that iDPs improved their insufficiency in DP properties potentially *via* intercompartmental interaction with KCs within the constructs.

### KC-iDP Constructs Responded to WNT, SHH, and EDA Agonists to Upregulate HF-Related Markers

To evaluate functional resemblances between KC-DP and KC-iDP constructs, a WNT activator alone or the mixture of WNT, SHH, and EDA activators were added to the culture of KC-iDP constructs. The resultant structures were morphologically comparable to non-treated KC-DP and KC-iDP constructs with analogous HF marker expression patterns (Supplementary Figure 8). A readily distinguishable HF-like bulb structure was reproduced in a representative WNT + SHH + EDA agonist mixture-treated KC-iDP construct (Supplementary Figure 8).

Intriguingly, KC-iDP constructs tended to express higher levels of HF keratin genes, *KRT33A*, *KRT82*, and *KRT25*, than those in KC-DP constructs. When treated by a WNT agonist, KC-iDP constructs significantly upregulated these HF keratin genes, which were further enhanced by the activation of WNT, SHH, and EDA signaling pathways (*p* < 0.05; Figure 6A). *WNT5A* was upregulated by the addition of WNT activator alone or by the combination of triple factors in KC-iDP constructs to the level in KC-DP constructs. *LEF1* expression was the most intense in WNT-treated KC-iDP constructs among all constructs examined, which was greater in triple factor-treated KC-iDP constructs than in non-treated KC-DP or KC-iDP constructs (*p* < 0.05; Figure 6A).

Further HF marker expression assessment in KC and DP/iDP compartment in the constructs was conducted by immune or enzyme histochemistry (Figure 6B). Immunoreactivity of KRT75, a marker of HF companion layer, was detected in the inner KC compartment of KC-DP constructs, which was more intense in KC-iDP constructs (Figure 6C). GATA3, a representative IRS marker (Chikh et al., 2007), was positively stained in KC-DP and KC-iDP constructs (Figure 6C). DKK4, which is expressed in the hair placode and the hair shaft and partially in IRS of HF (Fliniaux et al., 2008), was observed in inner KC layers in KC-DP and KC-iDP constructs (Figure 6B). Furthermore, SOX2, LEF1, and ALP, which were expressed in the bulb, especially in DPs (Supplementary Figure 5), were more strongly stained in iDPs of KC-iDP constructs compared to those in KC-DP constructs (Figure 6C).

These findings suggested that iDPs would be functionally sufficient to replace DPs in KC-DP constructs, at least, to reproduce HF characteristics examined in the aforementioned three-dimensional culture protocol.

## Discussion

The HF is a mini-organ consisting of multiple dynamically interacting epithelial and dermal components. Their anatomical relationships were crucial to elicit optimal EMIs to maintain the HF homeostasis represented by the hair cycle (Toyoshima et al., 2012; Abaci et al., 2018). Accordingly, microanatomical reproduction of HF architecture is pivotal to bioengineer structures fully reproducing HF architecture, let alone major functions (Abaci et al., 2018). Folliculogenic EMIs can partially be induced *in vitro* by the spheroid culture of HF-related epithelial and dermal cell mixtures (e.g., KCs and DP cells), which would not allow full recapitulation of three-dimensional (intercompartmental and directional) features of EMIs (Havlickova et al., 2009; Yen et al., 2010). Recent approaches took advantage of artificially assembling epithelial and mesenchymal components prior to *in vitro* or *in vivo* incubation *via* cell compartmentalization or molding to direct EMIs in a similar manner as in the HF (Toyoshima et al., 2012; Abaci et al., 2018). Adopting this strategy with substantial simplification, the methodology in the current study was invented.

Past studies demonstrated that cell aggregation ameliorated biological properties of cultured DP cells (Osada et al., 2007; Higgins et al., 2010; Kang et al., 2012; Ohyama et al., 2012; Higgins et al., 2013). The hair shaft has been reported to correlate with that of DP (Chi et al., 2013), suggesting that the magnitude of folliculogenic EMIs can be influenced by the size of regenerated DP cell aggregates. We initially attempted to generate larger DP cell aggregates; however, at least with our hands and the microenvironment of the assay developed, the central portion of DP aggregates with the size of human DPs tended to necrotize. Therefore, multiple smaller DP aggregates were generated and embedded as clusters in Matrigel. The DP aggregates did not completely fuse with each other but gradually gather together to form the assemblages during the 2-week culture. As the shape of the assemblages was hardly uniform among regenerated KC-DP constructs with the current approach, the magnitude of EMIs elicited in the KC-DP interface would be variable, which, together with the difference in biological properties of DP cells used for each experimentation, might account for the variance in HF-related keratin gene expression levels among KC-DP constructs. As HF markers, *WNT5A* and *LEF1*, were downregulated in KC-DP constructs than in control DP cells, the improvement in DP cell preparation is indispensable. Use of the dermal papilla activation culture condition (DPAC) (Ohyama et al., 2012) combined with an alternative aggregation protocol, e.g., the hanging drop culture (Higgins et al., 2013), may facilitate the preparation of more potent and larger DP cell spheroids replacing DP aggregates adopted in this study.

Using the protocol published by Toyoshima et al. as a reference, a nylon fiber was inserted into the center of KC-DP constructs. Originally, the investigators adopted this methodology for “*in vivo* transplantation of the bioengineered hair germ to prevent epithelial cyst formation and allow the bioengineered HF epithelium connected to that of the host animals leaving the hair canal for the emerging hair shaft” (Toyoshima et al., 2012). In the current study, the fiber is rather used to regulate the directionality of EMIs and maintain HF-like morphology by avoiding cell-autonomous migration/aggregation with resultant deformity of the *in vitro* regenerated constructs. In the assay developed, nylon fiber insertion is unfavorable as it vertically divides the KC-DP interface, leading to the split in EMIs. KC-DP constructs generated without a nylon fiber failed to sustain structural integrity, suggesting the necessity of some device to preserve morphological characteristics in the *in vitro* assay.

During the 2-week culture, KCs intercompartmentally differentiated or rearranged themselves to form multiple layers with a distinct histological characteristic and keratin expression pattern. As the constructs made of KCs and fibroblast aggregates failed to form stratified KC layers, EMIs between DP aggregates and adjacent KCs are speculated to have contributed to the formation of such layered structures. Cylindrical injection of KCs alone with the 2-week culture yielded KRT13 or KRT14 expressing outermost bilayers analogous to that observed in KC-DP constructs with central necrosis in the KC compartment (data not shown), suggesting that the microenvironment by itself, putatively *via* extracellular matrix contact or nutrition, pH, and hypoxia gradient, could also play roles in the recapitulation of HF-like keratin distribution in KC-DP constructs. Based on the change in KRT19 and KRT40 in KCs and VCAN and SMA expression in the dermal component, 2 weeks would be an optimal time for harvesting the culture product. At least with the current culture condition, further follicular differentiation by extending cultivation period cannot be expected. Histological examination of 4-week cultured KC-DP constructs detected robust intracompartmental necrosis. Incorporation of fibroblasts or vascular endothelial cells in Matrigel (Abaci et al., 2018) may extend the life span of the constructs. At the same time, such modifications complicate the protocol and can moderate the handiness of the assay.

Upregulation of HF-related keratins and mesenchymal markers by the addition of a WNT agonist or the mixture of WNT, SHH, and EDA agonists to the KC-DP construct culture implied an alternative approach to improve the assay by modulating the intensity of signaling pathways involved in HF morphogenesis. The concentrations for individual activators have been optimized for three-dimensional skin equivalent culture (Fukuyama et al., 2020), not for the current HF reconstitution assay. Other signaling pathways, represented by BMP and FGF signaling, plays pivotal roles in HF formation and regeneration (Rendl et al., 2008; Biggs et al., 2018) and therefore the addition of agonists of such signaling pathways to the activator mixture potentially enhance ameliorative effects on KC-DP constructs. The change in the content of culture supplements/additives minimally affects the complexity of the assay, and therefore, further optimization of the agonist mixture composition represents an important next step.

Unexpectedly, generation of iDPs morphologically analogous to DPs was only possible with WD39-derived iDPSCs. Less aggregative behavior observed in other hiPSC lines, together with the observation that non-induced hiPSCs failed to form spheroid in the adopted condition, suggested incomplete induction of DP properties in hiPSC lines other than WD39. Our past observation supports that the WD39 line is intrinsically mesenchymal-prone (Veraitch et al., 2013). This might have resulted in differential expression of cell adhesion molecules or extracellular matrices and enabled WD39 to form usable iDPs. Recent studies suggested roles of Hox genes and extracellular matrix in the determination of dermal cell phenotype, including that of DP cells (Driskell et al., 2013; Jiang et al., 2018; Yu et al., 2018). Thus, as a next step, it would be important to examine whether the expression of such potential determinants is different between WD39- and other hiPSC-derived iDPSCs to explain this observation, which emphasizes the importance of assessing the biological properties of hiPSC lines prior to downstream applications. WD39-derived iDPs moderately expressed DP markers. Low dyeability to cytoplasmic membrane dyes also distinguished iDPs from DPs. Such findings had implied functional inferiority of iDPs compared to DPs. Unexpectedly, HF-related keratin and WNT signaling gene expression levels were comparable to or more intense in KC-iDP constructs than those in KC-DP constructs. Moreover, markedly positive immunoreactivity of KRT40 could occasionally be observed in the KC-iDP construct. These observations suggested that iDPs intensified their DP properties to levels analogous to those in DPs putatively *via* the interaction with KCs *in vitro*.

The stepwise increase in HF-related keratin gene expression by a WNT agonist or the mixture of WNT, SHH, and EDA agonists was observed in the KC-iDP construct, which is also in favor of the acquisition of DP properties by iDPs *in vitro*. The magnitudes of upregulation by the addition of SHH and EDA activators were lesser in KC-iDP constructs compared to those generated with KCs and DPs. This might be explained by an intrinsically intense WNT activation state in KC-iDPs constructs as demonstrated by *LEF1* upregulation. Considering that the activation of WNT pathway alone and triple pathways did not result in major morphological or biochemical differences in KC-iDP constructs, additional modification to the culture condition, presumably *via* supplementation of extra signaling activators (e.g., SHH and BMP agonist), is mandated for further quality improvement of the KC-iDP construct.

We are aware of the limitations of the current study. Preparation of histological sections, especially frozen sections, was technically challenging because of the smallness and fragility of regenerated structures, which sometimes hampers accurate evaluation or comparisons. Manual reconstruction would not allow precisely consistent assembly of the constructs, potentially resulting in the variance in the size of the KC-DP or KC-iDP interface area, which could have affected the intensity of EMIs and result in differential expression of HF-related markers among the samples. Because of this inconsistency and the restriction in the amount of collectable samples, global gene expression analysis can hardly be conducted. The current protocol for DP induction can be applicable to selected hiPSC lines. However, these drawbacks also highlight the key elements indispensable for successful *in vitro* bioengineering of human miniorgans adopting hiPSCs: preassessment of starting materials, importance of induction and cultivation period, microenvironment, intrastructurally directed EMls, and sufficient activation of pivotal signaling pathways.

The methodology developed in this study can provide a less complicated *in vitro* platform to assess the functionalities of tested mesenchymal components to elicit folliculogenic EMIs in an architecture sketchily resembling HFs, by which the possibility of using hiPSC-derived cell subsets for HF bioengineering was implied. With further modifications to the current protocol and hiPSC usage, the necessity of human tissue-derived trichogenic cell subsets, especially DPs, for regenerative medicine and drug discovery for hair loss disorders can be reduced.

## Data Availability Statement

The raw data supporting the conclusions of this article will be made available by the authors, without undue reservation.

## Ethics Statement

The studies involving human participants were reviewed and approved by The Institutional Review Board of Kyorin University (Protocol Nos. H27-022, H28-131, and H29-116). The patients/participants provided their written informed consent to participate in this study.

## Author Contributions

MO designed the experiments. MF, AT, MK, YY, and MO performed the experiments and analyzed the data. HO supervised the project and provided materials and technical support. MF, HO, and MO drafted the manuscript. All authors contributed to the completion of the manuscript.

## Conflict of Interest

MO is a scientific advisor of Taisho Pharmaceutical Co. Ltd., Rhoto Pharmaceutical Co. Ltd., Pfizer R&D Inc., and Ili Lilly Japan K.K. and receives research grants from Shiseido Co. and Maruho Co. for the topics not related to this study. HO is a member of the scientific advisory boards of San Bio Co. Ltd., Eisai Co. Ltd., and Daiichi Sankyo Co. Ltd. The remaining authors declare that the research was conducted in the absence of any commercial or financial relationships that could be construed as a potential conflict of interest.

## Publisher’s Note

All claims expressed in this article are solely those of the authors and do not necessarily represent those of their affiliated organizations, or those of the publisher, the editors and the reviewers. Any product that may be evaluated in this article, or claim that may be made by its manufacturer, is not guaranteed or endorsed by the publisher.

## Supplementary Material

Supplementary Figure 1 Morphology of KC-DP constructs generated without nylon fibers. Despite some constructs maintained club-like morphology, most constructs lost their structural characteristics to form less organized cell aggregates. Scale bar: 200 μm.

Supplementary Figure 2 Histology of normal human hair follicle. The main body consisted of multiple keratinocyte layers with the dermal papilla at its proximal end. Scale bar: 200 μm.

Supplementary Figure 3 The constructs consisted of KCs and aggregated fibroblasts failed to form multiple KC layers. When human fibroblasts-aggregates were combined with condensed columlar keratinocytes (KCs), multiple KC layers were not clearly formed unlike those in KC-DP constructs. Scale bar: 200 μm.

Supplementary Figure 4 Morphological and histological findings of KC-DP constructs cultured for 4 weeks. The constructs were mainly consisted of necrotic cells and was surrounded by denucleated keratotic cells (arrowhead). Scale bar: 200 μm.

Supplementary Figure 5 Keratins and dermal papilla-related marker expression pattern in human hair follicles. Keratin (KRT)13 is expressed in IRS, while KRT14 is detected in ORS. KRT17 is expressed in the inner aspect of ORS, while KRT19 is diffusely detectable in ORS from the bulge to the bulb. Immunoreactivity of KRT40, is specifically observed in the hair shaft. KRT75 is expressed in the companion layer. GATA3 is detected in IRS. DKK4 is weakly expressed in the hair shaft and in the root sheaths. VIM is a mesenchymal marker expressed in the dermal portion of the HF. VCAN and SMA are expressed in DS and DP. LEF1, SOX2, and ALP can be detected in DP. ALP, alkaline phosphatase; DKK4, dickkopf 4; DP, dermal papilla; DS, dermal sheath; GATA3, GATA binding protein 3; IRS, inner root sheath; KRT, keratin; LEF1, lymphoid enhancer binding factor 1; ORS, outer root sheath; SMA, smooth muscle actin; SOX2, sex determining region Y-box 2; VCAN, versican; VIM, vimentin. Scale bar: 200 μm.

Supplementary Figure 6 WD39 hiPSCs failed to form cell Keratin aggregates in sphere formation. Unlike induced hiPSC-derived dermal papilla substituting cells, non-induced WD39 hiPSCs were unable to form spheres in cell aggregation protocol adopted. Scale bar: 100 μm.

Supplementary Figure 7 Morphological comparison of WD39-, 201B7-hiPSC, and RPC-hiPS771-2-derived dermal papilla substituting aggregates. Note that 201B7 hiPSC formed less condensed fragile spheres, while RPC-hiPS771-2-hiPSCs hardly formed aggregates. hiPSC, human induced pluripotent stem cells. Scale bar: 100 μm.

Supplementary Figure 8 Morphological, histological, and immunohistochemical analyses of KC-iDP constructs treated with a WNT agonist or the mixture of WNT, SHH, and EDA agonists. Gross morphology, histological characteristics, and immunohistological staining profiles of KC-iDP constructs were analogous to that of non-treated KC-iDP constructs presented in Figure 5. The main body was consisted of multiple KC layers showing respective positive immunoreactivity of KRT13, 14, 17, 19, and 40. iDP compartment was positive for VIM, VCAN, and SMA staining. KC, keratinocyte; KRT, keratin; iDP, human induced pluripotent stem cell-derived dermal papilla substituting cell aggregates; SMA, smooth muscle actin; VIM, vimentin; VCAN, versican Scale bar: 200 μm.

Supplementary Table 1 Primer sequences for real-time PCR.
