# Supplementary figures and images for: Human iPS Cell-Derived Cell Aggregates Exhibited Dermal Papilla Cell Properties in in vitro Three-Dimensional Assemblage Mimicking Hair Follicle Structures
Source: Front Cell Dev Biol. 2021 Aug 2;9:590333. doi: 10.3389/fcell.2021.590333 (PMC8365839; doi:10.3389/fcell.2021.590333)

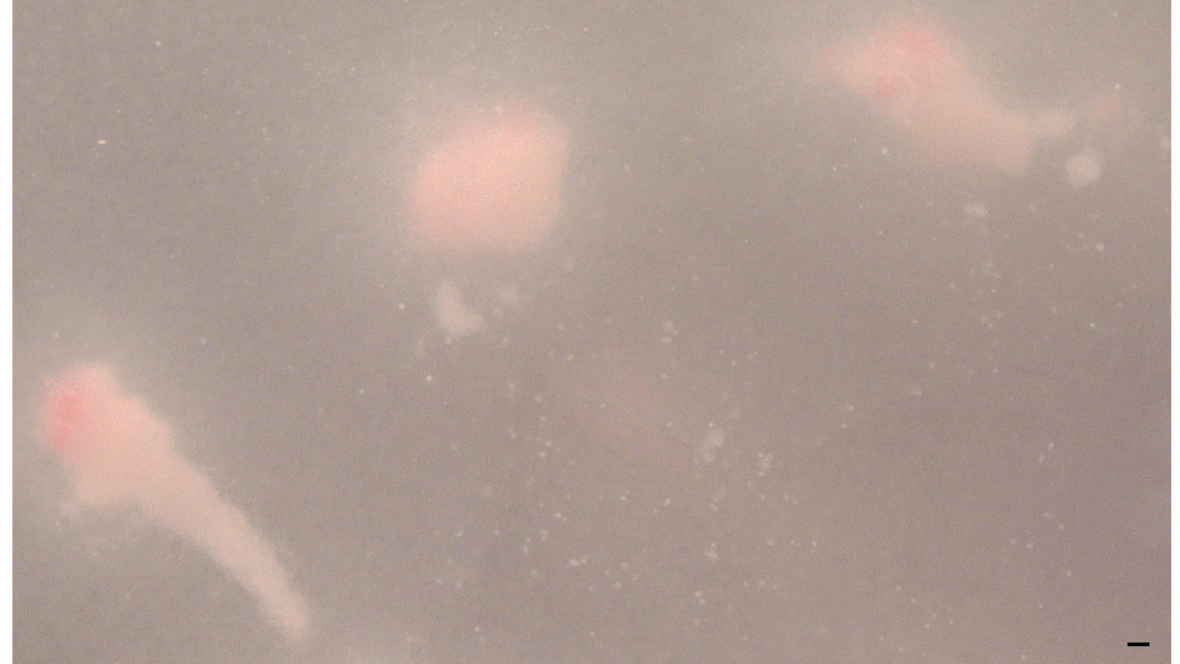

Supplement: Supplementary Figure 1 — Morphology of KC-DP constructs generated without nylon fibers. Despite some constructs maintained club-like morphology, most constructs lost their structural characteristics to form less organized cell aggregates. Scale bar: 200 μm. [file Image_1.TIF]

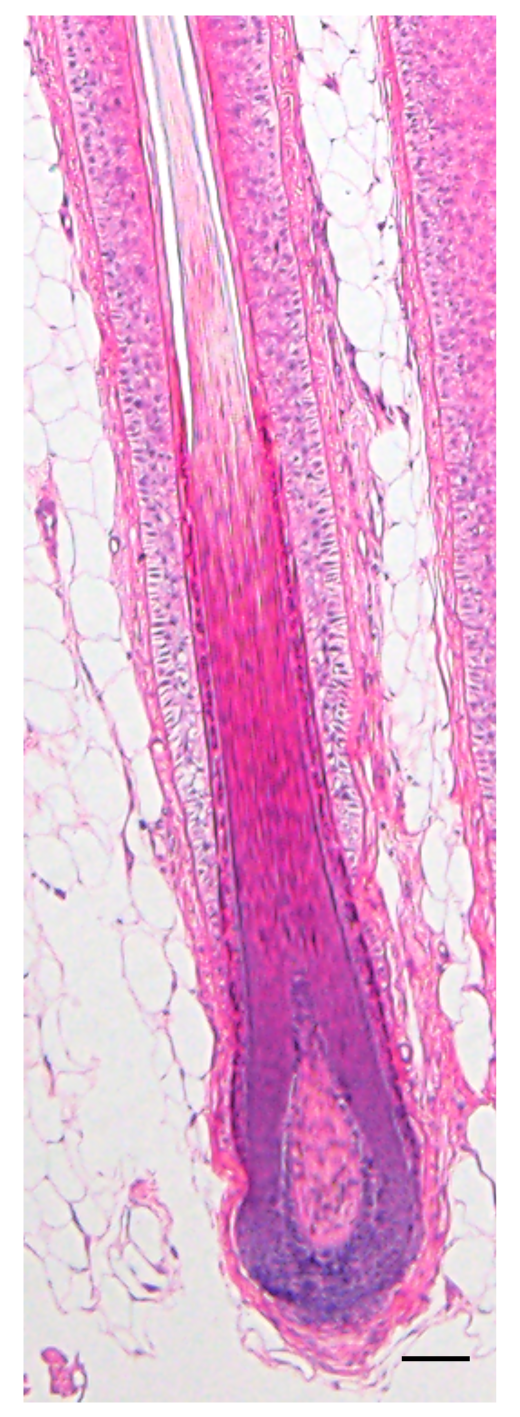

Supplement: Supplementary Figure 2 — Histology of normal human hair follicle. The main body consisted of multiple keratinocyte layers with the dermal papilla at its proximal end. Scale bar: 200 μm. [file Image_2.TIF]

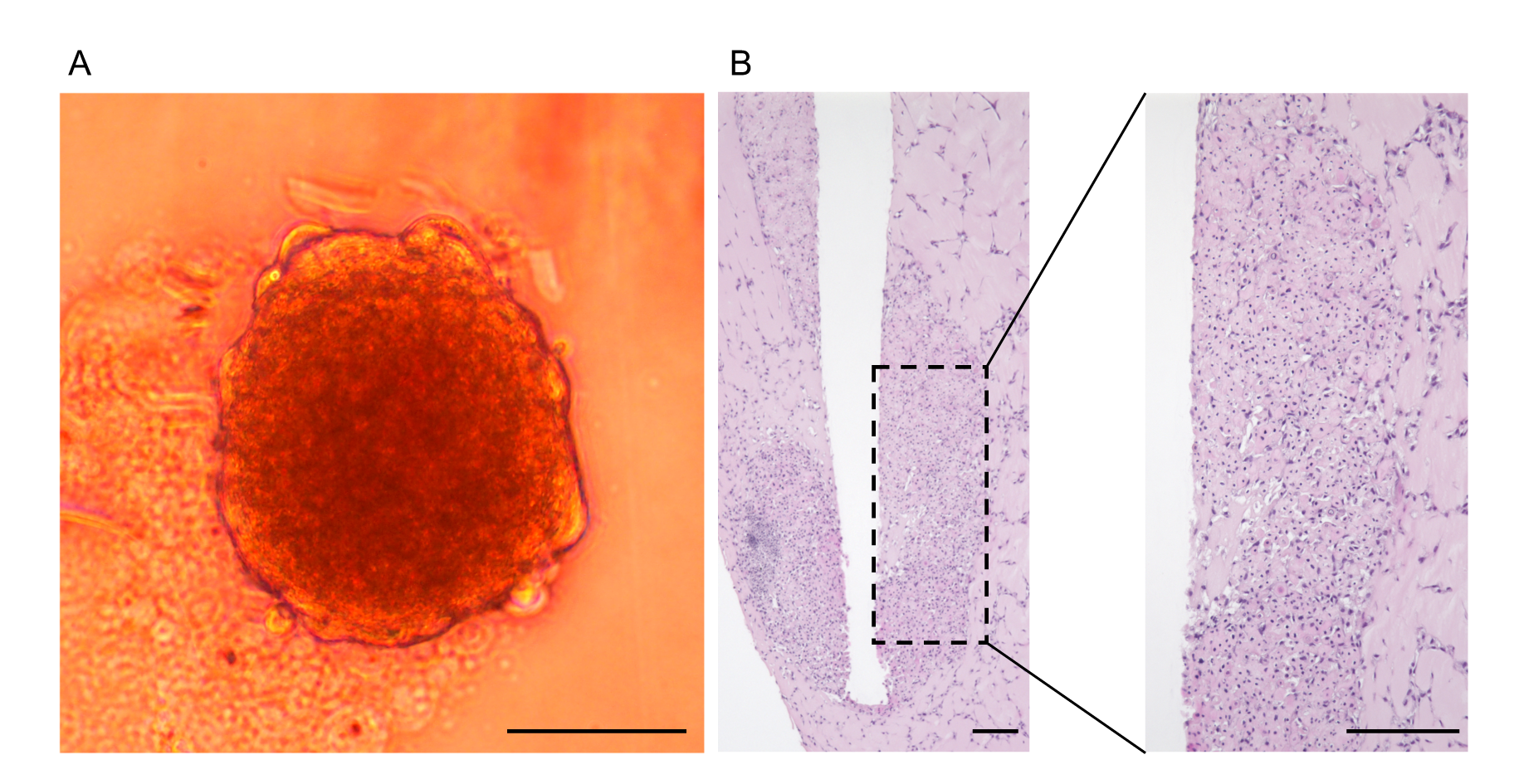

Supplement: Supplementary Figure 3 — The constructs consisted of KCs and aggregated fibroblasts failed to form multiple KC layers. When human fibroblasts-aggregates were combined with condensed columlar keratinocytes (KCs), multiple KC layers were not clearly formed unlike those in KC-DP constructs. Scale bar: 200 μm. [file Image_3.TIF]

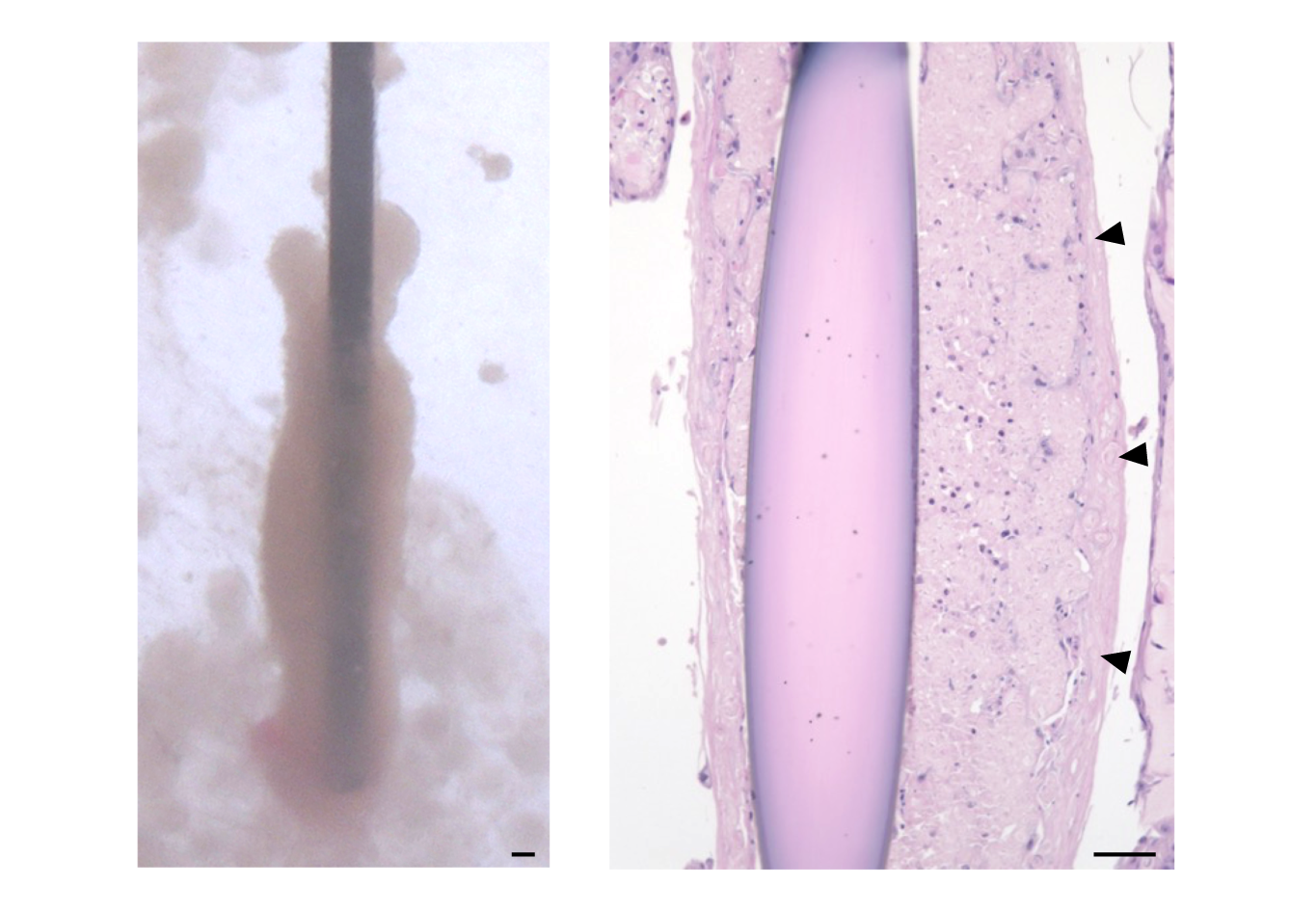

Supplement: Supplementary Figure 4 — Morphological and histological findings of KC-DP constructs cultured for 4 weeks. The constructs were mainly consisted of necrotic cells and was surrounded by denucleated keratotic cells (arrowhead). Scale bar: 200 μm. [file Image_4.TIF]

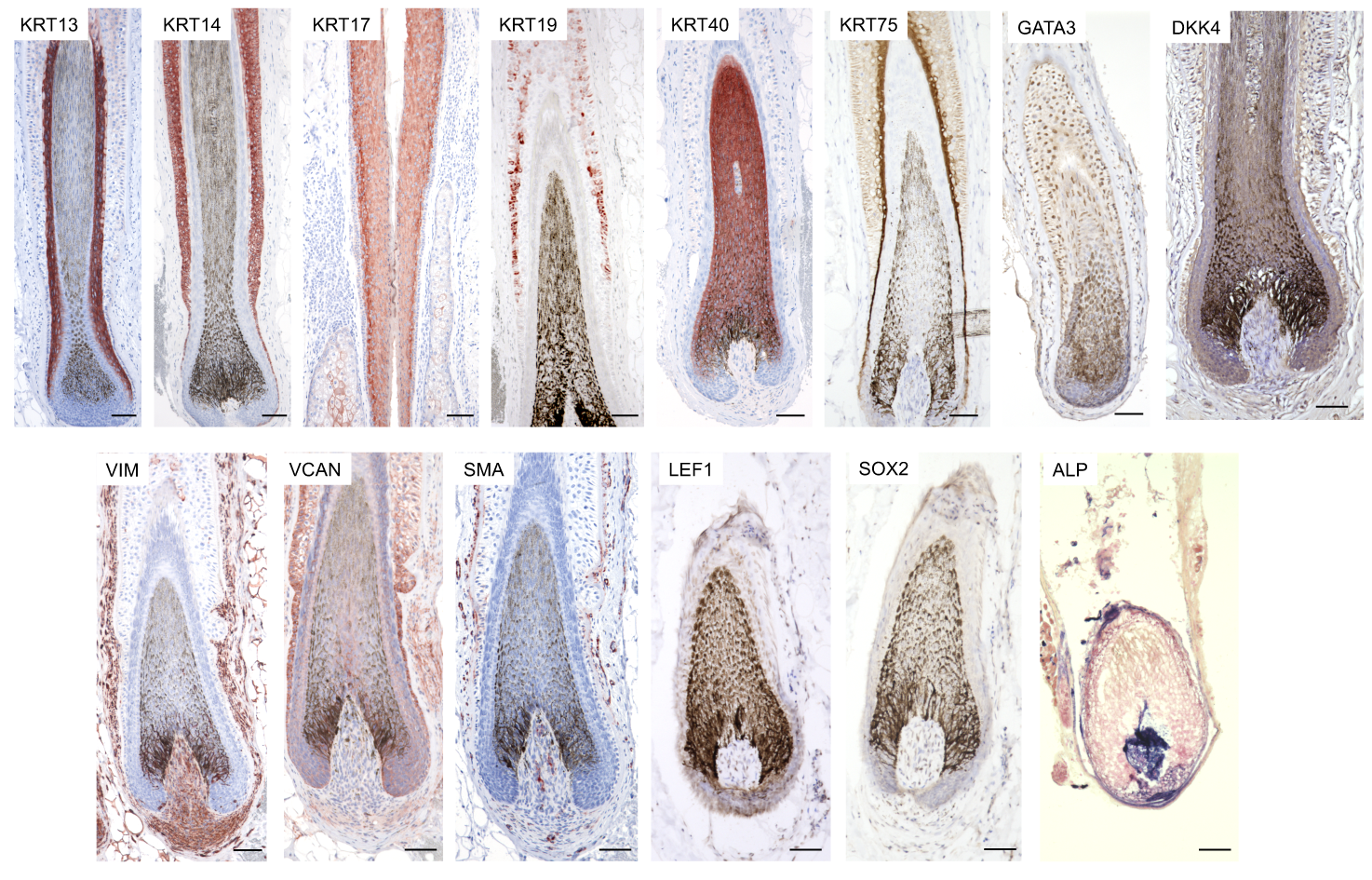

Supplement: Supplementary Figure 5 — Keratins and dermal papilla-related marker expression pattern in human hair follicles. Keratin (KRT)13 is expressed in IRS, while KRT14 is detected in ORS. KRT17 is expressed in the inner aspect of ORS, while KRT19 is diffusely detectable in ORS from the bulge to the bulb. Immunoreactivity of KRT40, is specifically observed in the hair shaft. KRT75 is expressed in the companion layer. GATA3 is detected in IRS. DKK4 is weakly expressed in the hair shaft and in the root sheaths. VIM is a mesenchymal marker expressed in the dermal portion of the HF. VCAN and SMA are expressed in DS and DP. LEF1, SOX2, and ALP can be detected in DP. ALP, alkaline phosphatase; DKK4, dickkopf 4; DP, dermal papilla; DS, dermal sheath; GATA3, GATA binding protein 3; IRS, inner root sheath; KRT, keratin; LEF1, lymphoid enhancer binding factor 1; ORS, outer root sheath; SMA, smooth muscle actin; SOX2, sex determining region Y-box 2; VCAN, versican; VIM, vimentin. Scale bar: 200 μm. [file Image_5.TIF]

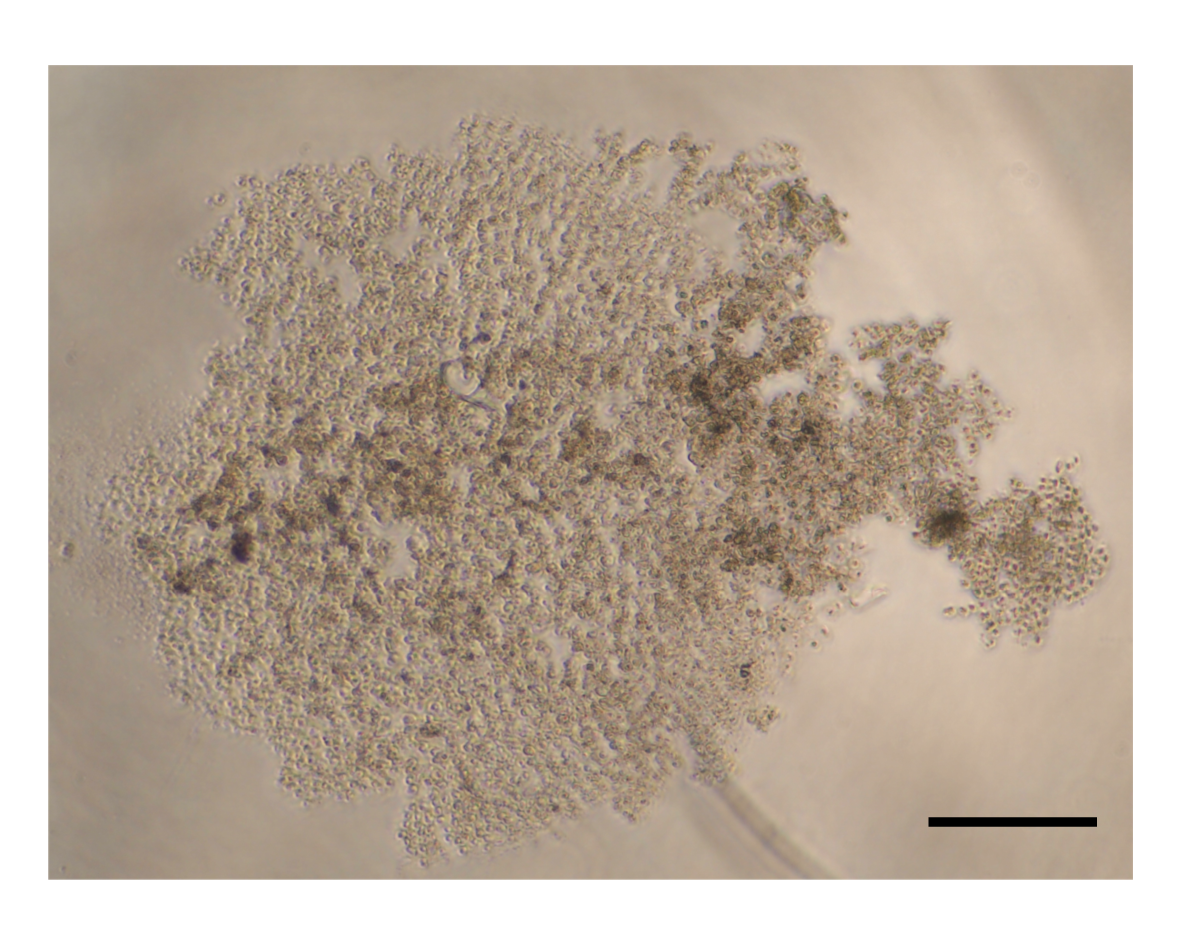

Supplement: Supplementary Figure 6 — WD39 hiPSCs failed to form cell Keratin aggregates in sphere formation. Unlike induced hiPSC-derived dermal papilla substituting cells, non-induced WD39 hiPSCs were unable to form spheres in cell aggregation protocol adopted. Scale bar: 100 μm. [file Image_6.TIF]

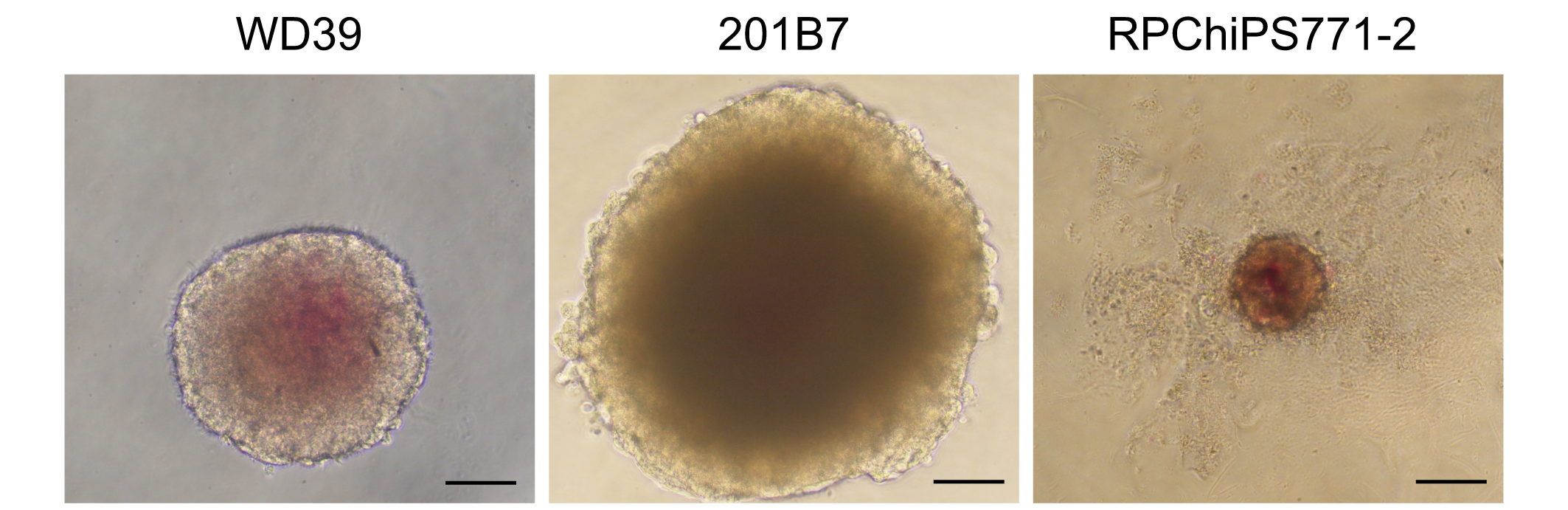

Supplement: Supplementary Figure 7 — Morphological comparison of WD39-, 201B7-hiPSC, and RPC-hiPS771-2-derived dermal papilla substituting aggregates. Note that 201B7 hiPSC formed less condensed fragile spheres, while RPC-hiPS771-2-hiPSCs hardly formed aggregates. hiPSC, human induced pluripotent stem cells. Scale bar: 100 μm. [file Image_7.TIF]

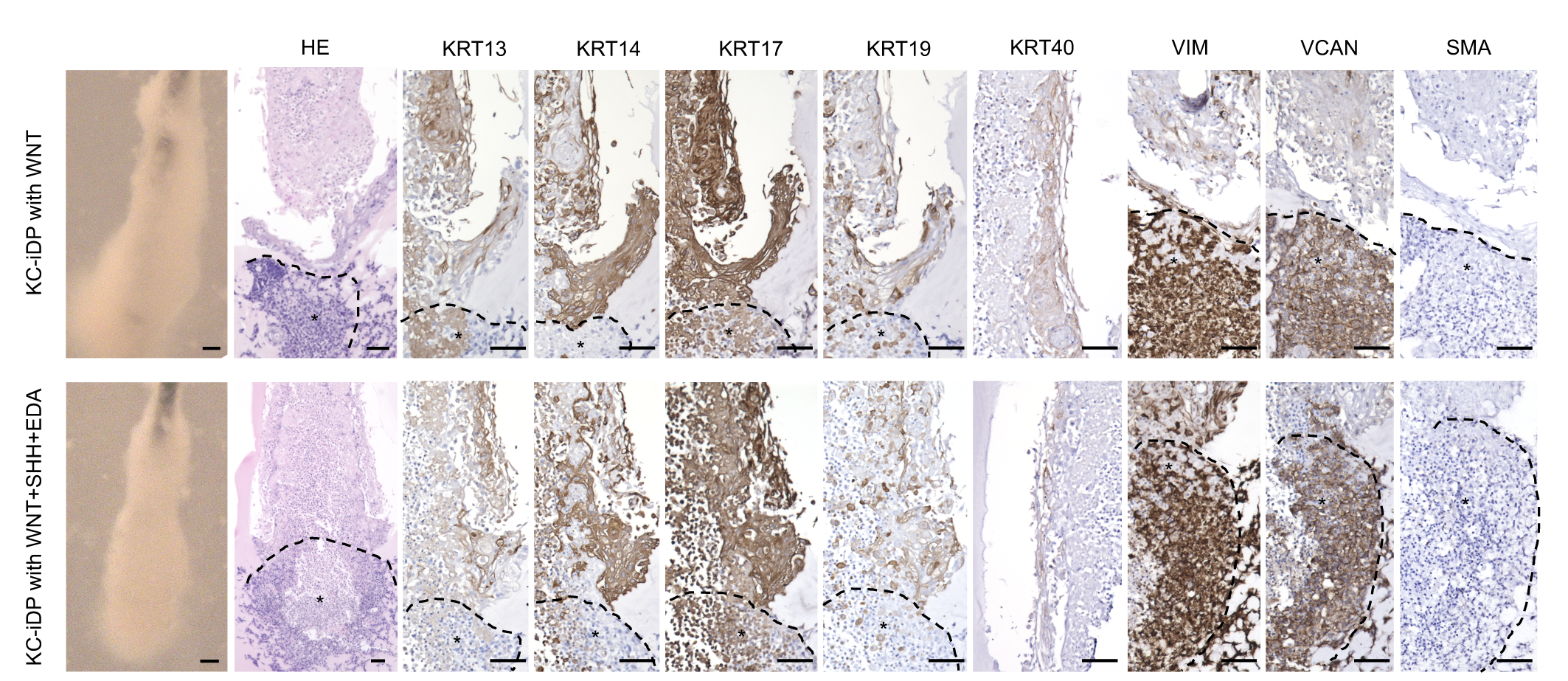

Supplement: Supplementary Figure 8 — Morphological, histological, and immunohistochemical analyses of KC-iDP constructs treated with a WNT agonist or the mixture of WNT, SHH, and EDA agonists. Gross morphology, histological characteristics, and immunohistological staining profiles of KC-iDP constructs were analogous to that of non-treated KC-iDP constructs presented in Figure 5. The main body was consisted of multiple KC layers showing respective positive immunoreactivity of KRT13, 14, 17, 19, and 40. iDP compartment was positive for VIM, VCAN, and SMA staining. KC, keratinocyte; KRT, keratin; iDP, human induced pluripotent stem cell-derived dermal papilla substituting cell aggregates; SMA, smooth muscle actin; VIM, vimentin; VCAN, versican Scale bar: 200 μm. [file Image_8.TIF]
