# Supplementary material for: Human iPS Cell-Derived Cell Aggregates Exhibited Dermal Papilla Cell Properties in in vitro Three-Dimensional Assemblage Mimicking Hair Follicle Structures
Source: Front Cell Dev Biol. 2021 Aug 2;9:590333. doi: 10.3389/fcell.2021.590333 (PMC8365839; doi:10.3389/fcell.2021.590333)
Supplement: Supplementary Table 1 — Primer sequences for real-time PCR. [file Table_1.DOC]

Table S1. Primer sequences for real-time PCR

| Gene | Primer sequences |
| --- | --- |
| KRT25 | (F) 5’-ATGTCTCTTCGACTTTCCAGTGC-3’ |
|  | (R) 5’-GCCACTTCCAATCCCTGAAAT-3’ |
| KRT33A | (F) 5’-TGCATGTGACAAGTCCACTG-3’ |
|  | (R) 5’-ACCCAAATGTGTTGCAAGGC-3’ |
| KRT82 | (F) 5’-TCACCCCTGTCACCATCAATG-3’ |
|  | (R) 5’-ATGCGAAACGGTTGTTGAGG-3’ |
| WNT5A | (F) 5’-TCCACCTTCCTCTTCACACTGA-3’ |
|  | (R) 5’-CGTGGCCAGCATCACATC-3’ |
| LEF1 | (F) 5’-CCCGATGACGGAAAGCAT-3’ |
|  | (R) 5’-TCGAGTAGGAGGGTCCCTTGT-3’ |
| ALPL | (F) 5’-ATTGACCACGGGCACCAT-3’ |
|  | (R) 5’-CTCCACCGCCTCATGCA-3’ |
| NOG | (F) 5’-CTGGTGGACCTCATCGAACA-3’ |
|  | (R) 5’-CGTCTCGTTCAGATCCTTTTCCT-3’ |
| SPRY4 | (F) 5’-TTGGTGCAGGGCATCTTCTAC-3’ |
|  | (R) 5’-GCGCAGGAGCCCTCATC-3’ |
| LRP4 | (F) 5’-GGCACAGCCACTAGGTTTTAACA-3’ |
|  | (R) 5’-GAAGGCCGAGGCAAGCA-3’ |

KRT25, keratin 25; KRT33A, keratin 33a; KRT82, keratin 82; WNT5A, Wnt family member 5A; LEF1, lymphoid enhancer binding factor 1; ALPL, alkaline phosphatase; NOG, noggin; SPRY4, sprouty homologs 4; LRP4, lipoprotein receptor-related protein 4
